# The method of lines extension for the analysis of multilayered graphene-loaded structures in cylindrical coordinates

**DOI:** 10.1038/s41598-022-17016-2

**Published:** 2022-07-26

**Authors:** Ali Mehrdadian, Keyvan Forooraghi, Mehri Ziaee Bideskan

**Affiliations:** Department of Electrical and Computer Engineering, Tarbiat Modares University, Tehran, 14115-194 Iran

**Keywords:** Electrical and electronic engineering, Physics

## Abstract

In this paper the extended method of lines (E-MoL) is proposed for the analysis of multilayer graphene-loaded three dimensional structures in cylindrical coordinates. Accordingly, the impedance and admittance matrices are defined as the ratios of the electric and magnetic fields at each plane of the stack. The impedance and admittance parameters are transformed from the input to the output of the structure through layers and interfaces, from which, the scattering parameters are extracted. It is assumed that there is an anisotropic graphene layer at the interface of two successive layers. The impedance and admittance transformations at the interfaces are extracted in the cylindrical coordinates. Then the impedance and admittance values at all planes of the stack and consequently, the scattering parameters of the whole structure are derived. To validate the presented method, two validation benchmarks are provided at the microwave frequency band. A circular waveguide and a coaxial cable loaded with graphene plates are analyzed and the results are compared with those of CST simulation software which show good accordance. It is observed that the E-MoL, as a semi-analytical semi-numerical method, is much more time-efficient than the CST software numerical procedure.

## Introduction

Graphene is a one-atom thick allotrope of carbon arranged in a hexagonal lattice with extraordinary properties^1^. This 2D material is a promising candidate in electronics and electromagnetics and has attracted the researchers’ attention all around the world in the recent years^2,3^. Nowadays, graphene sheets with dimensions up to 30 cm are fabricated, and this has led to the use of graphene in the new microwave and millimeter wave applications^4^. Graphene sheets have remarkable electric properties like controlling their surface conductivity through applying electromagnetic fields or DC voltages. This feature is used to design tunable electronic or electromagnetic devices^5,6^. New nano-sized electronic and electromagnetic components and devices such as antennas, flexible electronic devices, touch screens and high-speed transistors are examples of the states of the art in the graphene world. The unique properties of graphene are due to its gapless electronic band structure^7–10^.

Various methods have been used to analyze multilayer three-dimensional graphene-loaded structures. In^11^ per unit length equivalent circuits are proposed for the accurate characterization of propagation in different graphene-loaded waveguides. For that purpose, analytical expressions for the graphene conductivity as well as lumped circuit components are calculated through which the propagation of surface waves along spatial graphene waveguides is illustrated. The finite difference time domain (FDTD) method has the advantage of solving transient broadband problems in one simulation. In^12^, the FDTD method is exploited to investigate the electromagnetic interaction with graphene by treating it as a surface boundary condition and sampling the computational space. Recently, in^13^ the magnetized graphene is characterized by an anisotropic surface boundary condition using FDTD. Physically, the aim of the proposed method in^13^ is to incorporate magnetically-biased graphene formulation into electromagnetic wave scattering problems macroscopically by modeling the graphene with a conducting sheet. Li et al.^14^ introduce the discontinuous Galerkin time-domain (DGTD) method to characterize the transient response of the graphene from microwaves to terahertz frequencies. Having considered an infinite graphene sheet with its finite surface conductivity^14^, deals with resistive boundary conditions for the graphene. Moreover, the method of moments (MoM) is used for the diffraction analysis of graphene nanoribbons in^15^. The authors also try to model non-local effects of graphene by the MoM analysis. In^16^ graphene nanoribbons are analyzed using space-domain MoM with the restriction that the method is valid only for low wavenumbers. Lastly, the theory of characteristic modes is used to characterize the input impedance and efficiency of a plasmonic graphene-based antenna^17^. In^18,19^, based on integral equations governing the surface current density of magnetically biased graphene, an analytical approach is proposed to analyze an array of graphene ribbons.

The approaches mentioned above have advantages and disadvantages. Pure analytical methods are very time-effective but are able to solve only certain simple structures. On the other hand, fully numerical methods are capable of analyzing complex structures, but generally they are very time consuming. The method of lines (MoL) is a simple, fast, and accurate approach that solves an electromagnetic problem analytically in one coordinate direction and numerically in other coordinate directions^20–22^. This makes MoL especially appropriate to deal with general multilayered structures loaded with complicated boundy conditions of arbitrary shapes, where the wave propagation in the direction perpendicular to the layers stacking is treated analytically. In directions transverse to the layers the structure is discretized and is sloved numerically. MoL can be used to analyze 2D and 3D structures that require 1D and 2D discretizations, respectively^23^. The MoL enjoys the advantages of both analytical and numerical methods while avoiding the shortcomings of each method when modeling multilayered structures. In^24,25^, we have proposed MoL for the analysis of 2D structures including graphene plates in Cartesian coordinates. To verify the efficiency of the method, several 2D graphene-loaded structures such as microstrips, striplines and parallel-plate waveguides are presented and analyzed. In^26^, we extend the MoL for the analysis of graphene-loaded 3D structures in Cartesian coordinates. Besides, different graphene-based periodic structures again in Cartesian coordinates, are studied in^27^ using MoL.

In this paper the MoL is extended in cylindrical coordinates for the analysis of multilayered structures with an arbitrary number of layers loaded with graphene plates of arbitrary shapes. Here the graphene plates are treated as layers represented by surface boundary conditions. To do so, the impedance and admittance transformation formulas through a graphene plate are extracted. Having obtained the transformations, one can compute at every plane of the structure the impedance and admittance values, and subsequently the scattering parameters are derived. For the purpose of validation, the MoL results are compared with those of CST full wave simulation software which are in good accordance. In the first example, we assume a circular waveguide loaded with graphene sheets and perform a parametric analysis in terms of the graphene chemical potential for different numbers of plates. The second example deals with a coaxial cable loaded with annular graphene sheets. The proposed method is general and can be used to analyze generic 3D multilayered stackings in cylindrical coordinates with applications in absorbers and radiating structures. Note that the MoL is a pure classical theory which is applied to Maxwell’s equations and thus it is not able to model quantum mechanical phenomena such as Klein tunneling which occurs in graphene sheets^28,29^.

The rest of the paper is organized as follows: in “The method of lines in cylindrical coordinates” section, the E-MoL is presented in its general form for the analysis of multilayer graphene-loaded three-dimensional structures in the cylindrical coordinates. In “Validation of the proposed method” section, the numerical results concerning the reflection and transmission coefficients of a circular waveguide and a coaxial cable loaded with graphene plates are given as validation scenarios using the proposed theory and CST simulation software. Finally, in “Conclusion” section, the conclusions are provided.

## The method of lines in cylindrical coordinates

In this section, the generalized transmission line (GTL) equations are first given in cylindrical coordinates to analyze structures that require two-dimensional discretization. Then, the impedance and admittance transformation formulas in a homogenous layer are given. The presence of graphene at the interface of two adjacent layers will result in a new set of boundary conditions through which the impedance and admittance transformation formulas at the interface are extracted. As a result, the impedance and admittance values are derived in each layer of the multilayer structure. Having obtained all impedances and admittances, the electromagnetic fields all over the structure and subsequently the scattering parameters of the whole structure are achievable.

### The generalized transmission line equations in cylindrical coordinates

In what follows, we assume that the constitutive parameters are defined by:

1 $$\overleftrightarrow \upsilon_{r}=\left[\begin{array}{lll}{\upsilon }_{rr}& {\upsilon }_{r\varphi }& 0\\ {\upsilon }_{\varphi r}& {\upsilon }_{\varphi \varphi }& 0\\ 0& 0& {\upsilon }_{zz}\end{array}\right]=\left[\begin{array}{cc}\overleftrightarrow \upsilon_{rc}& 0\\ 0& {\upsilon }_{zz}\end{array}\right]\upupsilon =\upvarepsilon , \mu ,\upsigma $$

First, we present the GTL equations suitable for *z* direction propagation. The first and second equations of Ampere's law and Faraday's induction law result in^30^:

2 $$-j\left[\begin{array}{ll}{\varepsilon }_{\phi \phi }& {\varepsilon }_{\phi r}\\ {\varepsilon }_{r\phi }& {\varepsilon }_{rr}\end{array}\right]\left[\begin{array}{c}{E}_{\phi }\\ {E}_{r}\end{array}\right]=\frac{\partial }{\partial \overline{z} }\left[\begin{array}{c}-{\widetilde{H}}_{r}\\ {\widetilde{H}}_{\phi }\end{array}\right]+\left[\begin{array}{c} {D}_{\overline{r} }\\ -{\overline{r} }^{-1}{D}_{\phi }\end{array}\right]{\widetilde{H}}_{z}$$

3 $$-j\left[\begin{array}{cc} {\mu }_{rr}& -{\mu }_{r\phi }\\ -{\mu }_{\phi r}& {\mu }_{\phi \phi }\end{array}\right]\left[\begin{array}{c}-{\widetilde{H}}_{r}\\ {\widetilde{H}}_{\phi }\end{array}\right]=\frac{\partial }{\partial \overline{z} }\left[\begin{array}{c}{E}_{\phi }\\ {E}_{r}\end{array}\right]  - \left[ {\begin{array}{*{20}c} {\bar{r}^{{ - 1}} D_{\phi } }  \\    {D_{{\bar{r}}} }  \\ \end{array} } \right] {E}_{z}$$

in which the coordinates and dimensions are normalized to the free space wave number ${k}_{0}$ according to $\overline{z }={k}_{0}z$ and $\overline{r }={k}_{0}r$. Besides, the transverse field components in Eqs. (2) and (3) are arranged in a way that the inner product of the field vectors with the transverse field components is proportional to the Poynting vector in *z*-direction. Using the third equations of the Ampere’s Law and Faraday induction’s Law, the longitudinal field components ${\widetilde{H}}_{z}$ and ${E}_{z}$ are obtained as follows:

4 $${\widetilde{H}}_{z}=j{\mu }_{zz}^{-1}{\overline{r} }^{-1}\left[\begin{array}{cc}{D}_{\overline{r} }& -{D}_{\phi }\end{array}\right]\left[{E}^{z}\right].\quad {E}_{z}=-j{\varepsilon }_{zz}^{-1}{\overline{r} }^{-1} \left[ {\begin{array}{*{20}c}    {D_{\phi } } & {D_{{\bar{r}}} }  \\   \end{array} } \right] \left[{H}^{z}\right]$$

with transverse fields vectors being defined as $$\left[{E}^{z}\right]=\left[\begin{array}{c}\overline{r}{E }_{\phi }\\ {E}_{r}\end{array}\right]$$ and $$\left[{H}^{z}\right]=\left[\begin{array}{c}-{\widetilde{H}}_{r}\\ \overline{r}{\widetilde{H} }_{\phi }\end{array}\right]$$. Substituting Eq. (4) in Eq. (2) and Eq. (3), @@the relation between the transverse field components are obtained as^30^:

5 $$\frac{\partial }{{\partial \bar{z}}}\left[ {H^{z} } \right] =  - j\left[ {R_{E}^{z} } \right]\left[ {E^{z} } \right].~\quad ~R_{E}^{z}  = \left[ {\begin{array}{*{20}c}    {D_{{\bar{r}}} \mu _{{zz}}^{{ - 1}} \bar{r}^{{ - 1}} D_{{\bar{r}}}  + \varepsilon _{{\phi \phi }} \bar{r}^{{ - 1}} } & { - D_{{\bar{r}}} \mu _{{zz}}^{{ - 1}} \bar{r}^{{ - 1}} D_{\phi }  + \varepsilon _{{\phi r}} }  \\    { - D_{\phi } \mu _{{zz}}^{{ - 1}} \bar{r}^{{ - 1}} D_{{\bar{r}}}  + \varepsilon _{{r\phi }} } & {D_{\phi } \mu _{{zz}}^{{ - 1}} \bar{r}^{{ - 1}} D_{\phi }  + \varepsilon _{{rr}} \bar{r}}  \\   \end{array} } \right]$$

6 $$\frac{\partial }{\partial \overline{z} }\left[{E}^{z}\right]=-j\left[{R}_{H}^{z}\right]\left[{H}^{z}\right]. {R}_{H}^{z}=\left[\begin{array}{cc}{D}_{\phi }{\varepsilon }_{zz}^{-1}{\overline{r} }^{-1}{D}_{\phi }+{\mu }_{rr}\overline{r }& {D}_{\phi }{\varepsilon }_{zz}^{-1}{\overline{r} }^{-1}{D}_{\overline{r} }-{\mu }_{r\phi }\\ {D}_{\overline{r}}{\varepsilon  }_{zz}^{-1}{\overline{r} }^{-1}{D}_{\phi }-{\mu }_{\phi r}& {D}_{\overline{r}}{\varepsilon  }_{zz}^{-1}{\overline{r} }^{-1}{D}_{\overline{r} }+{\mu }_{\phi \phi }{\overline{r} }^{-1}\end{array}\right]$$

Then, we combine the two first-order differential equation systems in Eqs. (5) and (6) in order to obtain the second-order differential equations for the transverse electric or magnetic fields as:

7 $$\frac{{\partial }^{2}}{\partial {\overline{z} }^{2}}\left[{E}^{z}\right]-\left[{Q}_{E}^{z}\right]\left[{E}^{z}\right]=0. \left[{Q}_{H}^{z}\right]=-\left[{R}_{E}^{z}\right]\left[{R}_{H}^{z}\right]$$

8 $$\frac{{\partial }^{2}}{\partial {\overline{z} }^{2}}\left[{H}^{z}\right]-\left[{Q}_{H}^{z}\right]\left[{H}^{z}\right]=0. \left[{Q}_{E}^{z}\right]=-\left[{R}_{H}^{z}\right]\left[{R}_{E}^{z}\right]$$

These equations are called as wave equations. To solve these equations using MoL, the fields and their derivatives in transverse directions should be discretized.

### Discretization of the fields and solutions

**Figure 1:**
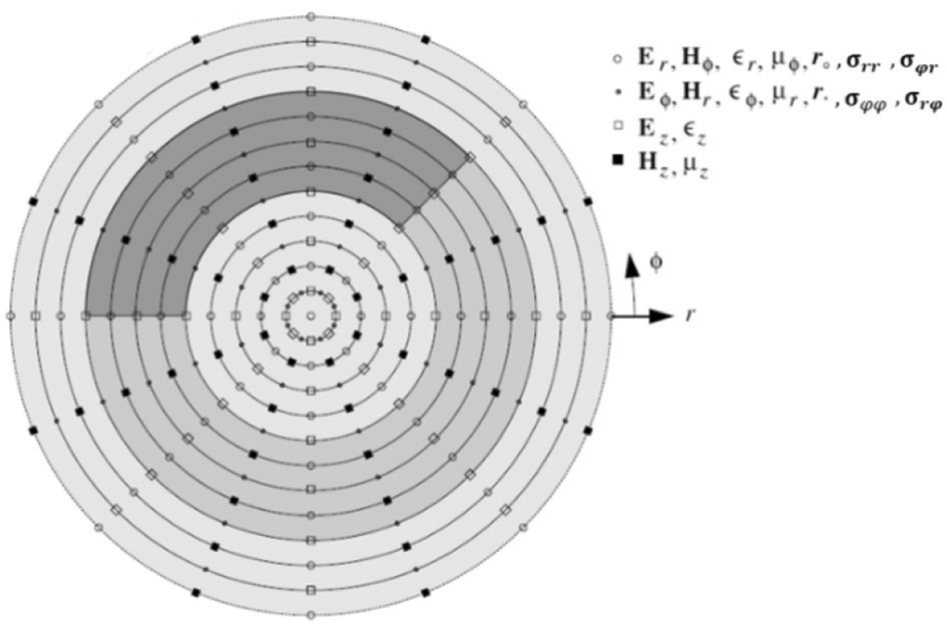
The discretization scheme in the cross section of a cylindrical waveguide with inhomogeneous layers.

In this section, we present the discretization of the fields components in the cross section as well as the solution of the GTL equations in the discretized form. The discretization scheme is shown in Fig. 1.

In what follows, the 2D discretization quantities are marked with a hat $(\widehat{ })$. The discretized field components are collected in vectors. To do so, the discretization is started from the waveguide’s center and continue in the radial direction. The discretized constitutive parameters and radii form diagonal matrices. The difference operators are replaced with central differences in radial and azimuthal directions and form the entries of matrices ${{\varvec{D}}}_{\overline{r} }^{\circ.\bullet}$ and ${{\varvec{D}}}_{\phi }^{\circ.\bullet}$, respectively. To obtain the difference operators in the 2D discretization scheme, we use the Kronecker product of difference matrix operators in radial and azimuthal directions, i.e. ${{\varvec{D}}}_{\overline{r} }^{\circ.\bullet}$ and ${{\varvec{D}}}_{\phi }^{\circ.\bullet}$, and unit matrices, as follows^30^:

9 $${\widehat{{\varvec{D}}}}_{\overline{r} }^{\circ.\bullet }={{\varvec{I}}}_{\phi }^{\circ.\bullet} \otimes{ {\varvec{D}}}_{\overline{r} }^{\circ.\bullet}. {\widehat{{\varvec{D}}}}_{\phi }^{\circ.\bullet }={{\varvec{D}}}_{\phi }^{\circ.\bullet} \otimes{ {\varvec{I}}}_{r}^{\circ.\bullet}$$

in which ${{\varvec{I}}}_{r}^{\circ.\bullet}$ and ${{\varvec{I}}}_{\phi }^{\circ.\bullet}$ are unit matrices of sizes ${N}_{r}^{\circ.\bullet}$ (the number of discretization points in radial direction) and ${N}_{\phi }^{\circ.\bullet}$(the number of discretization points in azimuthal direction), respectively. Besides, the difference matrix operators in the radial direction ${\widehat{{\varvec{D}}}}_{\overline{r} }^{\circ.\bullet}$ for the circle $\left( \circ \right)$ and dot $\left( \bullet \right)$ points are constructed using the Dirichlet (Neumann) and Neumann (Dirichlet) boundary conditions in case of magnetic (electric) walls, respectively. For ${\widehat{{\varvec{D}}}}_{\phi }^{\circ.\bullet}$, the periodic boundary condition is employed in case the complete discretization of the cross section in azimuthal direction is required. To continue with the discretization of GTL equations we define^30^:

10 $$\widehat{{\varvec{H}}}={\left[-{\widetilde{{\varvec{H}}}}_{r}^{t}. {\overline{{\varvec{r}}} }_{o}{\widetilde{{\varvec{H}}}}_{\phi }^{t}\right]}^{t}. \widehat{{\varvec{E}}}={\left[{\overline{{\varvec{r}}} }_{\bullet}{{\varvec{E}}}_{\phi }^{t}. {{\varvec{E}}}_{r}^{t}\right]}^{t}$$

and use the following abbreviations:

11 $${\widehat{\overline{{\varvec{\varepsilon}}}} }_{r}\equiv {\widehat{{\varvec{\varepsilon}}}}_{rr}{\widehat{\overline{{\varvec{r}}}} }_{o} {\widehat{\overline{{\varvec{\varepsilon}}}} }_{\phi }\equiv {\widehat{{\varvec{\varepsilon}}}}_{\phi \phi }{{\widehat{\overline{{\varvec{r}}}} }_{\bullet}}^{-1} {\widehat{\overline{{\varvec{\varepsilon}}}} }_{z}\equiv {\widehat{{\varvec{\varepsilon}}}}_{zz}{\widehat{\overline{{\varvec{r}}}} }_{\square }$$

12 $${\widehat{\overline{{\varvec{\mu}}}} }_{r}\equiv {\widehat{{\varvec{\mu}}}}_{rr}{\widehat{\overline{{\varvec{r}}}} }_{\bullet} {\widehat{\overline{{\varvec{\mu}}}} }_{\phi }\equiv {\widehat{{\varvec{\varepsilon}}}}_{\phi \phi }{{\widehat{\overline{{\varvec{r}}}} }_{o}}^{-1} {\widehat{\overline{{\varvec{\mu}}}} }_{z}\equiv {\widehat{{\varvec{\mu}}}}_{zz}{\widehat{\overline{{\varvec{r}}}} }_{\blacksquare}$$

As a result, Eqs. (5)–(8) are discretized as follows:

13 $$\frac{d}{d\overline{z}}\widehat{{\varvec{H}} }=-j{\widehat{{\varvec{R}}}}_{E}^{z}\widehat{{\varvec{E}}} \frac{d}{d\overline{z}}\widehat{{\varvec{E}} }=-j{\widehat{{\varvec{R}}}}_{H}^{z}\widehat{{\varvec{H}}}$$

14 $$\frac{{d}^{2}}{d{\overline{z} }^{2}}\widehat{{\varvec{H}}}-{\widehat{{\varvec{Q}}}}_{H}^{z}\widehat{{\varvec{H}}}=0 \frac{{d}^{2}}{d{\overline{z} }^{2}}\widehat{{\varvec{E}}}-{\widehat{{\varvec{Q}}}}_{E}^{z}\widehat{{\varvec{E}}}=0$$

in which

15 $$\user2{\hat{R}}_{E}^{z}  = \left[ {\begin{array}{*{20}c}    {\widehat{{\user2{\bar{\varepsilon }}}}_{\phi }  - ~\widehat{{\user2{\bar{D}}}}_{{\bar{r}}}^{{ \bullet t}} \widehat{{\user2{\bar{\mu }}}}_{z}^{{ - 1}} ~\widehat{{\user2{\bar{D}}}}_{{\bar{r}}}^{ \bullet } } & {\user2{\hat{\varepsilon }}_{{\phi r}}  + ~\widehat{{\user2{\bar{D}}}}_{{\bar{r}}}^{{ \bullet t}} \widehat{{\user2{\bar{\mu }}}}_{z}^{{ - 1}} ~\widehat{{\user2{\bar{D}}}}_{\phi }^{o} }  \\    {~\widehat{{\user2{\bar{D}}}}_{\phi }^{{ot}} \widehat{{\user2{\bar{\mu }}}}_{z}^{{ - 1}} ~\widehat{{\user2{\bar{D}}}}_{{\bar{r}}}^{ \bullet }  + \user2{\hat{\varepsilon }}_{{r\phi }} } & {\user2{\hat{\varepsilon }}_{r}  - ~\widehat{{\user2{\bar{D}}}}_{\phi }^{{ot}} \widehat{{\user2{\bar{\mu }}}}_{z}^{{ - 1}} ~\widehat{{\user2{\bar{D}}}}_{\phi }^{o} }  \\   \end{array} } \right]$$

16 $$\user2{\hat{R}}_{H}^{z}  = \left[ {\begin{array}{*{20}c}    {\widehat{{\user2{\bar{\mu }}}}_{r}  - ~\widehat{{\user2{\bar{D}}}}_{\phi }^{{ \bullet t}} \widehat{{\user2{\bar{\varepsilon }}}}_{z}^{{ - 1}} ~\widehat{{\user2{\bar{D}}}}_{\phi }^{ \bullet } } & {\user2{\hat{M}}_{{r\phi }}  - ~\widehat{{\user2{\bar{D}}}}_{\phi }^{{ \bullet t}} \user2{\hat{\varepsilon }}_{z}^{{ - 1}} ~\widehat{{\user2{\bar{D}}}}_{{\bar{r}}}^{o} }  \\    {\user2{\hat{M}}_{{\phi r}}  - ~\widehat{{\user2{\bar{D}}}}_{{\bar{r}}}^{{ot}} \widehat{{\user2{\bar{\varepsilon }}}}_{z}^{{ - 1}} ~\widehat{{\user2{\bar{D}}}}_{\phi }^{ \bullet } } & {\user2{\hat{\mu }}_{\phi }  - ~\widehat{{\user2{\bar{D}}}}_{{\bar{r}}}^{{ot}} \widehat{{\user2{\bar{\varepsilon }}}}_{z}^{{ - 1}} ~\widehat{{\user2{\bar{D}}}}_{{\bar{r}}}^{o} }  \\   \end{array} } \right]$$

17 $${\widehat{{\varvec{Q}}}}_{E}^{z}=-{\widehat{{\varvec{R}}}}_{H}^{z}{\widehat{{\varvec{R}}}}_{E}^{z}. {\widehat{{\varvec{Q}}}}_{H}^{z}=-{\widehat{{\varvec{R}}}}_{E}^{z}{\widehat{{\varvec{R}}}}_{H}^{z}$$

In Eqs. (15) and (16), $\widehat{\mathbf{M}}$ represents the interpolation matrices detailed in the following sections. Since the fields components are discretized in different positions they cannot be added or subtracted directly. Hence, interpolation matrices are used to compute the field components in the correct positions.

Now we are going to solve Eq. (14). For this purpose, we define the electric and magnetic fields as $\widehat{{\varvec{H}}}={{\varvec{T}}}_{H}\widehat{\overline{{\varvec{H}}} }$ and $\widehat{{\varvec{E}}}={{\varvec{T}}}_{E}\widehat{\overline{{\varvec{E}}} }$, in which ${{\varvec{T}}}_{E}$ and ${{\varvec{T}}}_{{\varvec{H}}}$ correspond to the electric and magnetic field distributions and are defined as the eigenvectors of ${\widehat{{\varvec{Q}}}}_{E}^{z}$ and ${\widehat{{\varvec{Q}}}}_{H}^{z}$, respectively. Besides,$\widehat{\overline{{\varvec{E}}} }$ and $\widehat{\overline{{\varvec{H}}} }$ are the electric and magnetic field amplitude vectors.

Introducing $\widehat{{\varvec{H}}}={{\varvec{T}}}_{H}\widehat{\overline{{\varvec{H}}} }$ and $\widehat{{\varvec{E}}}={{\varvec{T}}}_{E}\widehat{\overline{{\varvec{E}}} }$ into Eq. (14), one obtains:

18 $$\frac{{d}^{2}}{d{\overline{z} }^{2}}\widehat{\overline{{\varvec{H}}} }-{{\varvec{\Gamma}}}_{H}^{2}\widehat{\overline{{\varvec{H}}} }=0.\boldsymbol{ }\boldsymbol{ }\boldsymbol{ }\boldsymbol{ }\boldsymbol{ }\boldsymbol{ }\boldsymbol{ }\frac{{d}^{2}}{d{\overline{z} }^{2}}\widehat{\overline{{\varvec{E}}} }-{{\varvec{\Gamma}}}_{E}^{2}\widehat{\overline{{\varvec{E}}} }=0$$

with

19 $${{\varvec{T}}}_{H}^{-1}{{\varvec{Q}}}_{H}{{\varvec{T}}}_{H}={{\varvec{\Gamma}}}_{H}^{2}. {{\varvec{T}}}_{E}^{-1}{{\varvec{Q}}}_{E}{{\varvec{T}}}_{E}={{\varvec{\Gamma}}}_{E}^{2}$$

Since ${\widehat{{\varvec{Q}}}}_{E}^{z}={\widehat{{\varvec{Q}}}}_{H}^{zt}$, the following relations hold between the eigenvector and eigenvalue matrices of ${\widehat{{\varvec{Q}}}}_{E}^{z}$ and ${\widehat{{\varvec{Q}}}}_{H}^{z}$:

20 $${{\varvec{\Gamma}}}_{E}^{2}={{\varvec{\Gamma}}}_{H}^{2}={{\varvec{\Gamma}}}_{ }^{2}=-{{\varvec{\beta}}}^{2}. {{\varvec{T}}}_{E}={\widehat{{\varvec{R}}}}_{H}{{\varvec{T}}}_{H}{{\varvec{\beta}}}_{H}^{-1}. {{\varvec{T}}}_{H}={\widehat{{\varvec{R}}}}_{E}{{\varvec{T}}}_{E}{{\varvec{\beta}}}_{E}^{-1}$$

The general solution of Eq. (18) can be given as forward- and backward-propagating waves:

21 $$\widehat{\overline{{\varvec{F}}} }={\widehat{\overline{{\varvec{F}}}} }_{f}+{\widehat{\overline{{\varvec{F}}}} }_{b}={e}^{-j{\varvec{\beta}}\overline{z}}{\varvec{A} }+{e}^{+j{\varvec{\beta}}\overline{z}}{\varvec{B} }$$

where $\widehat{\overline{{\varvec{F}}} }=\widehat{\overline{{\varvec{E}}}}\boldsymbol{ }\mathrm{or}\boldsymbol{  }\widehat{\overline{{\varvec{H}}} }$. Also, due to the normalization introduced in Eq. (20), the characteristic impedance and admittance matrices are equal to identity matrices.

Having known the electric and magnetic fields in each plane according to Eq. (21), the relation between the electric and magnetic fields at two different planes with distance *d*, are obtained as^30^:

22 $$\left[\begin{array}{c}{\widehat{\overline{{\varvec{H}}}} }_{A}\\ -{\widehat{\overline{{\varvec{H}}}} }_{B}\end{array}\right]=\left[\begin{array}{cc}{\overline{{\varvec{y}}} }_{1}& {\overline{{\varvec{y}}} }_{2}\\ {\overline{{\varvec{y}}} }_{2}& {\overline{{\varvec{y}}} }_{1}\end{array}\right]\left[\begin{array}{c}{\widehat{\overline{{\varvec{E}}}} }_{A}\\ {\widehat{\overline{{\varvec{E}}}} }_{B}\end{array}\right]. \left[\begin{array}{c}{\widehat{\overline{{\varvec{E}}}} }_{A}\\ {\widehat{\overline{{\varvec{E}}}} }_{B}\end{array}\right]=\left[\begin{array}{cc}{\overline{{\varvec{z}}} }_{1}& {\overline{{\varvec{z}}} }_{2}\\ {\overline{{\varvec{z}}} }_{2}& {\overline{{\varvec{z}}} }_{1}\end{array}\right]\left[\begin{array}{c}{\widehat{\overline{{\varvec{H}}}} }_{A}\\ -{\widehat{\overline{{\varvec{H}}}} }_{B}\end{array}\right]$$

with

23 $$ \begin{aligned} & \overline{\user2{y}}_{1} = \widehat{{\overline{\user2{Y}}}}_{0} /{\text{tanh}}\left( {{\hat{\mathbf{\Gamma }}}\overline{d}} \right)\quad { }\overline{\user2{y}}_{2} = - \widehat{{\overline{\user2{Y}}}}_{0} /{\text{sinh}}\left( {{\hat{\mathbf{\Gamma }}}\overline{d}} \right) \\ & \overline{\user2{z}}_{1} = \widehat{{\overline{\user2{Z}}}}_{0} /{\text{tanh}}\left( {{\hat{\mathbf{\Gamma }}}\overline{d}} \right)\quad { }\overline{\user2{z}}_{2} = \widehat{{\overline{\user2{Z}}}}_{0} /{\text{sinh}}\left( {{\hat{\mathbf{\Gamma }}}\overline{d}} \right) \\ \end{aligned} $$

On the other hand, the electric and magnetic fields at each plane are related to each other through impedance/admittance matrices as follows:

24 $${\widehat{\overline{{\varvec{H}}}} }_{A.B}={\widehat{\overline{{\varvec{Y}}}} }_{A.B}{\widehat{\overline{{\varvec{E}}}} }_{A.B}.\quad  {\widehat{\overline{{\varvec{E}}}} }_{A.B}={\widehat{\overline{{\varvec{Z}}}} }_{A.B}{\widehat{\overline{{\varvec{H}}}} }_{A.B}$$

Consequently, the impedance and admittance transformation formulas between planes A and B are as follows:

25 $${\widehat{\overline{{\varvec{Y}}}} }_{A}={\overline{{\varvec{y}}} }_{1}-{\overline{{\varvec{y}}} }_{2}{\left({\overline{{\varvec{y}}} }_{1}+{\widehat{\overline{{\varvec{Y}}}} }_{B}\right)}^{-1}{\overline{{\varvec{y}}} }_{2}.\, {\widehat{\overline{{\varvec{Z}}}} }_{A}={\overline{{\varvec{z}}} }_{1}-{\overline{{\varvec{z}}} }_{2}{({\overline{{\varvec{z}}} }_{1}+{\widehat{\overline{{\varvec{Z}}}} }_{B})}^{-1}{\overline{{\varvec{z}}} }_{2}$$

### Impedance and admittance transformation formulas at the graphene interface

**Figure 2:**
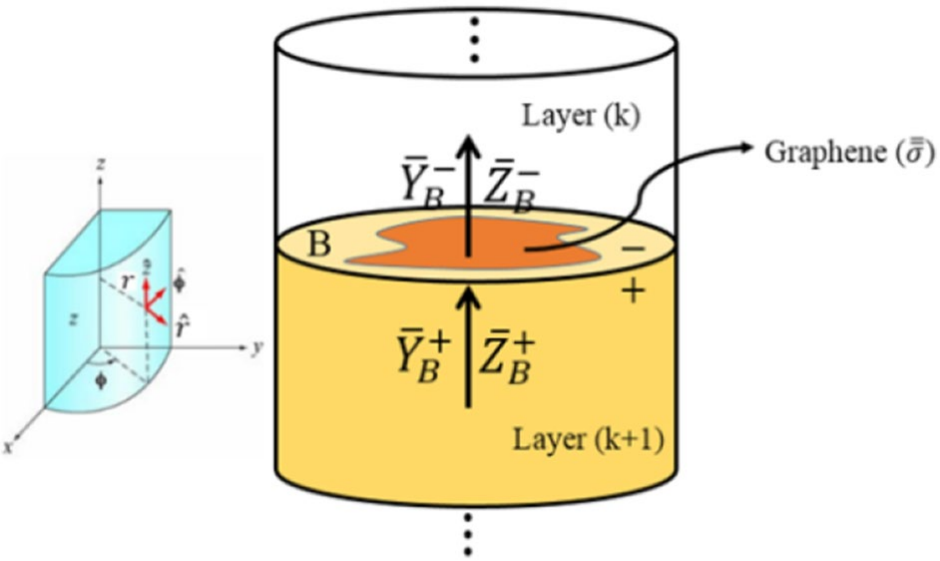
graphene-loaded multilayer structure.

To analyze the whole structure, it is necessary to be able to pass through the graphene plates at the interface of two adjacent layers by transforming the impedances and admittances through the graphene interface. As shown in Fig. 2, we assume that there is a graphene plate between layers $k$ and $k+1$. This common interface is denoted by the letter *B*. Besides, the two sides of this interface are distinguished by + and − indices.

As shown in Fig. 2, the graphene is placed in $xy$ *o*r equivalently $r\varphi$ plane in which the GTL equations are discretized. Due to the semi-analytical nature of MoL, the equations are solved analytically in $z$ direction. For this reason, the analytical formulas must be extracted for the impedance and admittance transformation in this direction. To do so, we must relate the admittance (impedance) on both sides of the graphene interface, i.e. ${\overline{\mathrm{Y}} }_{B}^{+}$ and ${\overline{\mathrm{Y}} }_{B}^{-}$ (${\overline{\mathrm{Z}} }_{B}^{+}$ and ${\overline{\mathrm{Z}} }_{B}^{-})$. The electric current components on the graphene plate are defined by

26 $$\left[\begin{array}{c}{J}_{x}\\ {J}_{y}\end{array}\right]=\left[\begin{array}{c}{\sigma }_{xx}{E}_{x}-{\sigma }_{yx}{E}_{y}\\ {\sigma }_{xy}{E}_{x}+{\sigma }_{yy}{E}_{y}\end{array}\right]=\left[\begin{array}{cc}{\sigma }_{xx}& -{\sigma }_{yx}\\ {\sigma }_{xy}& {\sigma }_{yy}\end{array}\right]\left[\begin{array}{c}{E}_{x}\\ {E}_{y}\end{array}\right]=\overline{\overline{\sigma }}\left[\begin{array}{c}{E}_{x}\\ {E}_{y}\end{array}\right]$$

This equation must be transformed to the cylindrical coordinates system, which will be performed as below. The transverse electric field components must be continuous at the interface. Hence,

27 $${\left[\begin{array}{c}{E}_{r}\\ {E}_{\varphi }\end{array}\right]}^{{B}^{+}}={\left[\begin{array}{c}{E}_{r}\\ {E}_{\varphi }\end{array}\right]}^{{B}^{-}}={\left[\begin{array}{c}{E}_{r}\\ {E}_{\varphi }\end{array}\right]}^{B }={\overrightarrow{E}}_{B}$$

The transverse magnetic field components in cylindrical coordinates at $z=B$ are defined as

28 $${\overrightarrow{H}}_{{B}^{+}}={H}_{{rB}^{+}}\widehat{r}+{H}_{\varphi {B}^{+}}\widehat{\varphi }. \quad {\overrightarrow{H}}_{{B}^{-}}={H}_{{rB}^{-}}\widehat{r}+{H}_{\varphi {B}^{-}}\widehat{\varphi }$$

The boundary conditions for the magnetic field components at plane $B$ are

29 $$\widehat{z}\times \left({H}_{r{B}^{+}}\widehat{r}+{H}_{\varphi {B}^{+}}\widehat{\varphi }-{H}_{r{B}^{-}}\widehat{r}-{H}_{\varphi {B}^{-}}\widehat{\varphi }\right)={\overrightarrow{J}}_{B}=\overline{\overline{\sigma }}{\overrightarrow{E}}_{B}$$

Then, transformation of ${\overrightarrow{J}}_{B}$ defined by Eq. (26) into cylindrical coordinates results in

30 $$\widehat{z}\times \left({H}_{r{B}^{+}}\widehat{r}+{H}_{\varphi {B}^{+}}\widehat{\varphi }-{H}_{r{B}^{-}}\widehat{r}-{H}_{\varphi {B}^{-}}\widehat{\varphi }\right)={\overrightarrow{J}}_{B}=\left[\begin{array}{cc}{\sigma }_{\varphi \varphi }& {\sigma }_{\varphi r}\\ {\sigma }_{r\varphi }& {\sigma }_{rr}\end{array}\right]\left[\begin{array}{c}{E}_{\varphi B}\\ {E}_{rB}\end{array}\right]$$

where the graphene conductivity tensor is obtained in the cylindrical coordinates as

31 $$\left[\begin{array}{cc}{\sigma }_{rr}& {\sigma }_{r\varphi }\\ {\sigma }_{\varphi r}& {\sigma }_{\varphi \varphi }\end{array}\right]=\left[\begin{array}{cc}\mathrm{cos}\varphi & \mathrm{sin}\varphi \\ -\mathrm{sin}\varphi & \mathrm{cos}\varphi \end{array}\right]\left[\begin{array}{cc}{\sigma }_{xx}& -{\sigma }_{xy}\\ {\sigma }_{xy}& {\sigma }_{xx}\end{array}\right]\left[\begin{array}{cc}\mathrm{cos}\varphi & -\mathrm{sin}\varphi \\ \mathrm{sin}\varphi & \mathrm{cos}\varphi \end{array}\right]$$

Then, the $\widehat{r}$ and $\widehat{\varphi }$ components in Eq. (30) are separated as follows

32 $${H}_{r{B}^{+}}={H}_{r{B}^{-}}+{\sigma }_{\varphi r}{E}_{rB}+{\sigma }_{\varphi \varphi }{E}_{\varphi B}. \quad -{H}_{\varphi {B}^{+}}=-{H}_{\varphi {B}^{-}}+{\sigma }_{rr}{E}_{rB}+{\sigma }_{r\varphi }{E}_{\varphi B}$$

Defining the electric and magnetic field components in cylindrical coordinates as

33 $${E}_{ }=\left[\begin{array}{c}{\overline{r}E }_{\varphi }\\ {E}_{r}\end{array}\right] {H}_{ }={\eta }_{0}\left[\begin{array}{c}-{H}_{r}\\ {\overline{r}H }_{\varphi }\end{array}\right]$$

one can rewrite Eq. (32) in the vector form:

34 $${\eta }_{0}\left[\begin{array}{c}-{H}_{r{B}^{+}}\\ \overline{r}{H }_{\varphi {B}^{+}}\end{array}\right]={\eta }_{0}\left[\begin{array}{c}-{H}_{r{B}^{-}}\\ \overline{r}{H }_{\varphi {B}^{-}}\end{array}\right]+{\eta }_{0}\left[\begin{array}{cc}-\frac{1}{\overline{r}}{\sigma  }_{\varphi \varphi }& -{\sigma }_{\varphi r}\\ -{\sigma }_{r\varphi }& -\overline{r}{\sigma  }_{rr}\end{array}\right]\left[\begin{array}{c}{\overline{r}E }_{\varphi B}\\ {E}_{rB}\end{array}\right]$$

35 $${H}_{{B}^{+}}={H}_{{B}^{-}}+{\eta }_{0}\left[\begin{array}{cc}-\frac{1}{\overline{r}}{\sigma  }_{\varphi \varphi }& -{\sigma }_{\varphi r}\\ -{\sigma }_{r\varphi }& -\overline{r}{\sigma  }_{rr}\end{array}\right]{E}_{B}$$

In this step, we need to discretize the field components and graphene parameters in their appropriate places as shown in Fig. 1. The discretized form of Eq. (32) is:

36 $$-{\eta }_{0}{\mathbf{H}}_{r{B}^{+}}=-{\eta }_{0}{\mathbf{H}}_{r{B}^{-}}-{\eta }_{0}\frac{1}{\overline{{\varvec{r}}}}{{{\varvec{\upsigma}} }_{\varphi \varphi }}\overline{{\varvec{r}}}{\mathbf{E} }_{\varphi B}-{\eta }_{0}{{{\varvec{\upsigma}}}_{\varphi r}}{\mathbf{E}}_{rB}$$

37 $${\eta }_{0}\overline{{\varvec{r}}}{\mathbf{H} }_{\varphi {B}^{+}}={\eta }_{0}\overline{{\varvec{r}}}{\mathbf{H} }_{\varphi {B}^{-}}-{\eta }_{0}{{{\varvec{\upsigma}}}_{r\varphi }}\overline{{\varvec{r}}}{\mathbf{E} }_{\varphi B}-{\eta }_{0}\overline{{\varvec{r}}}{{{\varvec{\upsigma}} }_{rr}}{\mathbf{E}}_{rB}$$

As mentioned earlier, the field components, for example ${E}_{r}$ and ${E}_{\varphi }$ are discretized in different positions and hence we cannot add or subtract them directly to obtain ${H}_{r}.$ Hence, we need to use interpolation matrices to calculate the fields in the appropriate position. Accordingly, the discretized version of Eq. (34) can be expressed as

38 $$\left[\begin{array}{c}-{\eta }_{0}{\mathbf{H}}_{r{B}^{+}}\\ {\eta }_{0}\overline{{\varvec{r}}}{\mathbf{H} }_{\varphi {B}^{+}}\end{array}\right]=\left[\begin{array}{c}-{\eta }_{0}{\mathbf{H}}_{r{B}^{-}}\\ {\eta }_{0}\overline{{\varvec{r}}}{\mathbf{H} }_{\varphi {B}^{-}}\end{array}\right]+\left[\begin{array}{cc}-{\eta }_{0}\frac{1}{\overline{{\varvec{r}}}}{{{\varvec{\upsigma}} }_{\varphi \varphi }}& -{\eta }_{0}{\widehat{\mathbf{M}}}_{1}{{{\varvec{\upsigma}}}_{\varphi r}}\\ -{\eta }_{0}{\widehat{\mathbf{M}}}_{2}{{{\varvec{\upsigma}}}_{r\varphi }}& -{\eta }_{0}\overline{{\varvec{r}}}{{{\varvec{\upsigma}} }_{rr}}\end{array}\right]\left[\begin{array}{c}\overline{{\varvec{r}}}{\mathbf{E} }_{\varphi B}\\ {\mathbf{E}}_{rB}\end{array}\right]$$

${\widehat{\mathbf{M}}}_{1}$ interpolates ${\mathbf{E}}_{r}$ at ° points and calculates its corresponding values at • points. Similarly, ${\widehat{\mathbf{M}}}_{2}$ interpolates ${\mathbf{E}}_{\varphi }$ at • points and calculates its corresponding values at ° points.

According to Eq. (33), we rewrite Eq. (38) as

39 $${\widehat{\mathbf{H}}}_{{B}^{+}}={\widehat{\mathbf{H}}}_{{B}^{-}}+\left[{\varvec{\sigma}}\right]{\widehat{\mathbf{E}}}_{B}. \left[{\varvec{\sigma}}\right]=\left[\begin{array}{cc}-{\eta }_{0}\frac{1}{\overline{{\varvec{r}}}}{{{\varvec{\upsigma}} }_{\varphi \varphi }}& -{\eta }_{0}{\widehat{\mathbf{M}}}_{1}{{{\varvec{\upsigma}}}_{\varphi r}}\\ -{\eta }_{0}{\widehat{\mathbf{M}}}_{2}{{{\varvec{\upsigma}}}_{r\varphi }}& -{\eta }_{0}\overline{{\varvec{r}}}{{{\varvec{\upsigma}} }_{rr}}\end{array}\right]$$

Substituting $\widehat{{\varvec{H}}}={{\varvec{T}}}_{H}\widehat{\overline{{\varvec{H}}} }$ and $\widehat{{\varvec{E}}}={{\varvec{T}}}_{E}\widehat{\overline{{\varvec{E}}} }$ in Eq. (39) results in

40 $${\widehat{\mathbf{T}}}_{H}{\widehat{\overline{\mathbf{H}}} }_{{B}^{+}}={\widehat{\mathbf{T}}}_{H}{\widehat{\overline{\mathbf{H}}} }_{{B}^{-}}+\left[{\varvec{\sigma}}\right]{\widehat{\mathbf{T}}}_{E}{\widehat{\overline{\mathbf{E}}} }_{B}$$

Multiplying Eq. (40) by ${\widehat{\mathbf{T}}}_{H}^{-1}$, we obtain

41 $${\widehat{\overline{\mathbf{H}}} }_{{B}^{+}}={\widehat{\overline{\mathbf{H}}} }_{{B}^{-}}+{\widehat{\mathbf{T}}}_{H}^{-1}\left[{\varvec{\sigma}}\right]{\widehat{\mathbf{T}}}_{E}{\widehat{\overline{\mathbf{E}}} }_{B}$$

Moreover, according to Eq. (24) the relation between the electric and magnetic fields is calculated by ${\widehat{\overline{{\varvec{H}}}} }_{{B}^{-}}={\widehat{\overline{{\varvec{Y}}}} }_{{B}^{-}}{\widehat{\overline{{\varvec{E}}}} }_{B}$, substituting it in Eq. (41) results in

42 $${\widehat{\overline{\mathbf{H}}} }_{{B}^{+}}=\left({\widehat{\overline{\mathbf{Y}}} }_{{B}^{-}}+{\widehat{\mathbf{T}}}_{H}^{-1}\left[{\varvec{\sigma}}\right]{\widehat{\mathbf{T}}}_{E}\right){\widehat{\overline{\mathbf{E}}} }_{B}$$

Finally, the admittance matrix at $z={B}^{+}$ is derived as

43 $${\widehat{\overline{\mathbf{Y}}} }_{{B}^{+}}={\widehat{\overline{\mathbf{Y}}} }_{{B}^{-}}+{\widehat{\mathbf{T}}}_{H}^{-1}\left[{\varvec{\sigma}}\right]{\widehat{\mathbf{T}}}_{E}$$

Thus, transformation of admittance at graphene interface will be done using Eq. (43). Note that if the graphene does not cover the whole cross-section, Eq. (43) can still be used for admittance transformation. However, in those parts of the cross section where graphene is absent, the conductivity parameters are set to zero.

### Calculation of the scattering parameters

#### Reflection coefficient derivation

Starting from the output of the multilayer structure and using the formulas provided for impedance and admittance transformation through layers and interfaces, the input impedance and admittance can be obtained. Usually the load impedance is considered as the output wave impedance of the output waveguide which is normalized to be the identity matrix, i.e. ${{\varvec{Z}}}_{0}={\varvec{I}}$. At the input of the structure there is an input waveguide with a wave impedance ${\overline{\mathbf{Z}} }_{0}^{in}$ or a wave admittance ${\overline{\mathbf{Y}} }_{0}^{in}$.

Using the admittance transformation formulas, the input admittance ${\overline{\mathbf{Y}} }_{A}$ is calculated. By dividing the electric field into two parts, the forward and backward propagation components at the input of the waveguide are obtained as

44 $${\overline{\mathbf{H}} }_{A}={\overline{\mathbf{Y}} }_{A}\left({\overline{\mathbf{E}} }_{Af}+{\overline{\mathbf{E}} }_{Ab}\right)={\overline{\mathbf{Y}} }_{0}^{in}\left({\overline{\mathbf{E}} }_{Af}-{\overline{\mathbf{E}} }_{Ab}\right)$$

Consequently, for the scattering parameter ${\mathbf{S}}_{11}$ we have

45 $${\mathbf{S}}_{11}={\overline{\mathbf{E}} }_{Ab}{{\overline{\mathbf{E}} }_{Af}}^{-1}={\left({\overline{\mathbf{Y}} }_{0}^{in}+{\overline{\mathbf{Y}} }_{A}\right)}^{-1}\left({\overline{\mathbf{Y}} }_{0}^{in}-{\overline{\mathbf{Y}} }_{A}\right)$$

If the modes are ordered properly, the first element in the ${\mathbf{S}}_{11}$ matrix gives the fandamental mode reflection coefficient.

#### Transmission coefficient derivation

In order to calculate the transmission coefficient, the field coupled to the output port should be divided into the injected field at the input port. To do this, the fields should be calculated at the whole structure and they must be transformed in a numerically stable manner. The general solution of the wave equation in the Cartesian coordinates can be written as Eq. (21). The indices $f$ and $b$ denote the forward and backward propagations, respectively. The electric and magnetic fields are written as

46 $${\overline{\mathbf{H}} }^{\left(k\right)}={\overline{\mathbf{H}} }_{f}^{\left(k\right)}+{\overline{\mathbf{H}} }_{b}^{\left(k\right)}.\quad {\overline{\mathbf{E}} }^{\left(k\right)}={\overline{\mathbf{E}} }_{f}^{\left(k\right)}+{\overline{\mathbf{E}} }_{b}^{\left(k\right)}$$

The forward and backward parts are connected by the characteristic impedance ${\overline{\mathbf{Z}} }_{0}^{\left(k\right)}$

47 $${\overline{\mathbf{E}} }_{f}^{\left(k\right)}={\overline{\mathbf{Z}} }_{0}^{\left(k\right)}{\overline{\mathbf{H}} }_{f}^{\left(k\right)}.\quad {\overline{\mathbf{E}} }_{b}^{\left(k\right)}=-{\overline{\mathbf{Z}} }_{0}^{\left(k\right)}{\overline{\mathbf{H}} }_{b}^{\left(k\right)}$$

Then, the forward and backward parts can be obtained using the total fields

48 $${\overline{\mathbf{E}} }_{f}^{\left(k\right)}=\frac{1}{2}\left({\overline{\mathbf{E}} }^{\left(k\right)}+{\overline{\mathbf{Z}} }_{0}^{\left(k\right)}{\overline{\mathbf{H}} }^{\left(k\right)}\right).\quad {\overline{\mathbf{E}} }_{b}^{\left(k\right)}=\frac{1}{2}\left({\overline{\mathbf{E}} }^{\left(k\right)}-{\overline{\mathbf{Z}} }_{0}^{\left(k\right)}{\overline{\mathbf{H}} }^{\left(k\right)}\right)$$

49 $${\overline{\mathbf{H}} }_{f}^{\left(k\right)}=\frac{1}{2}\left({\overline{\mathbf{H}} }^{\left(k\right)}+{\overline{\mathbf{Y}} }_{0}^{\left(k\right)}{\overline{\mathbf{E}} }^{\left(k\right)}\right).\quad {\overline{\mathbf{H}} }_{b}^{\left(k\right)}=\frac{1}{2} \left({\overline{\mathbf{H}} }^{\left(k\right)}-{\overline{\mathbf{Y}} }_{0}^{\left(k\right)}{\overline{\mathbf{E}} }^{\left(k\right)}\right)$$

Using the definition of the impedance at any arbitrary plane, the above equations are written as

50 $${\overline{\mathbf{E}} }_{f}^{\left(k\right)}=\frac{1}{2}\left(\overline{{\varvec{I}} }+{\overline{\mathbf{Z}} }_{0}^{\left(k\right)}{\overline{\mathbf{Z}} }^{\left(k\right)-1}\right){\overline{\mathbf{E}} }^{\left(k\right)}.\quad {\overline{\mathbf{E}} }_{b}^{\left(k\right)}=\frac{1}{2}\left(\overline{{\varvec{I}} }-{\overline{\mathbf{Z}} }_{0}^{\left(k\right)}{\overline{\mathbf{Z}} }^{\left(k\right)-1}\right){\overline{\mathbf{E}} }^{\left(k\right)}$$

51 $${\overline{\mathbf{H}} }_{f}^{\left(k\right)}=\frac{1}{2}\left(\overline{{\varvec{I}} }+{\overline{\mathbf{Y}} }_{0}^{\left(k\right)}{\overline{\mathbf{Y}} }^{\left(k\right)-1}\right){\overline{\mathbf{H}} }^{\left(k\right)}.\quad {\overline{\mathbf{H}} }_{b}^{\left(k\right)}=\frac{1}{2}\left(\overline{{\varvec{I}} }-{\overline{\mathbf{Y}} }_{0}^{\left(k\right)}{\overline{\mathbf{Y}} }^{\left(k\right)-1}\right){\overline{\mathbf{H}} }^{\left(k\right)}$$

Assume that the field at the plane A is known. To avoid numerical instability, the following steps should be taken in order to calculate the fields at the plane B:

1. Using Eqs. (50) and (51), the vectors ${\overline{\mathbf{E}} }_{fA}^{\left(k\right)}$ and ${\overline{\mathbf{H}} }_{fA}^{\left(k\right)}$ are computed from the vectors ${\overline{\mathbf{E}} }_{A}^{\left(k\right)}$ and ${\overline{\mathbf{H}} }_{A}^{\left(k\right)}$.
2. Using the vectors ${\overline{\mathbf{E}} }_{fA}^{\left(k\right)}$ and ${\overline{\mathbf{H}} }_{fA}^{\left(k\right)}$, the vectors ${\overline{\mathbf{E}} }_{fB}^{\left(k\right)}$ and ${\overline{\mathbf{H}} }_{fB}^{\left(k\right)}$ are calculated as
  52 $${\overline{\mathbf{E}} }_{fB}^{\left(k\right)}={e}^{-{\varvec{\Gamma}}{\overline{d} }_{k}}{\overline{\mathbf{E}} }_{fA}^{\left(k\right)}.\quad {\overline{\mathbf{H}} }_{fB}^{\left(k\right)}={e}^{-{\varvec{\Gamma}}{\overline{d} }_{k}}{\overline{\mathbf{H}} }_{fA}^{\left(k\right)}$$
3. The total field at the plane B is computed as
  53 $${\overline{\mathbf{E}} }_{B}^{\left(k\right)}=2{\left(\overline{{\varvec{I}} }+{\overline{\mathbf{Z}} }_{0}^{\left(k\right)}{{\overline{\mathbf{Z}} }_{B}^{\left(k\right)-1}}^{ }\right)}^{-1}{\overline{\mathbf{E}} }_{fB}^{\left(k\right)}.\quad {\overline{\mathbf{H}} }_{B}^{\left(k\right)}={\overline{\mathbf{Y}} }_{B}^{\left(k\right)}{\overline{\mathbf{E}} }_{B}^{\left(k\right)}$$

**Figure 3:**
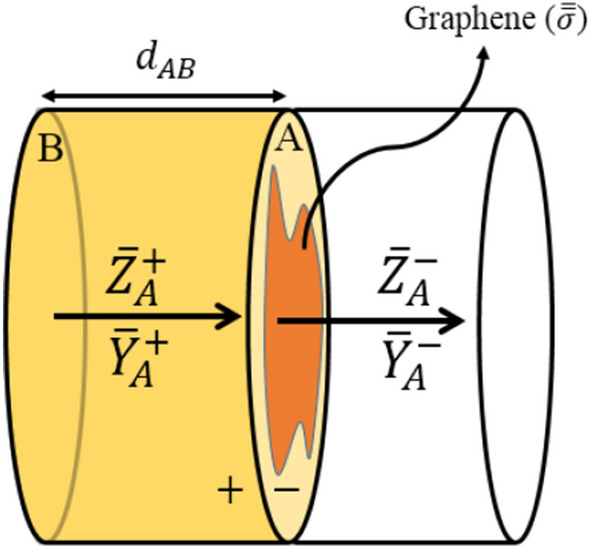
Two-layered structure with a graphene plate at the interface.

Then, supposing that the graphene plate is placed between the layers, the transmission coefficient is obtained. First, we assume that a graphene plate is located between the two layers of the structure, as shown in Fig. 3.

The values of the admittances $\left({\overline{{\varvec{Y}}} }_{{A}^{+}}. {\overline{{\varvec{Y}}} }_{{A}^{-}}\right)$ and impedances $\left({\overline{{\varvec{Z}}} }_{{A}^{+}}. {\overline{{\varvec{Z}}} }_{{A}^{-}}\right)$ shown in Fig. 3, are determined by the aforementioned admittance and impedance transformation formula Eq. (43). Also, the values ${\overline{{\varvec{Y}}} }_{B}$ and ${\overline{{\varvec{Z}}} }_{B}$ are calculated from Eq. (25). Assuming that these values are known, the transformation coefficient ${{\varvec{S}}}_{21}$ is derived. Using Eq. (53), the total field at this plane is written as follows

54 $${\overline{\mathbf{E}} }_{{A}^{+}}=2{\left(\overline{{\varvec{I}} }+{\overline{{\varvec{Z}}} }_{{A}^{+}}^{-1}\right)}^{-1}{\overline{\mathbf{E}} }_{f{A}^{+}}$$

The electric field at the interface of the plane A is continuous and hence ${\overline{\mathbf{E}} }_{{A}^{+}}={\overline{\mathbf{E}} }_{{A}^{-}}$. Due to normalization, the impedance and admittance values at the plane ${A}^{-}$ are equal to the characteristic impedance of the waveguide ${\overline{{\varvec{Z}}} }_{{{A}^{-}}}={\overline{{\varvec{Y}}} }_{{{A}^{-}}}=\mathbf{I}$. From Eq. (48), the values of the forward ${\overline{\mathbf{E}} }_{f{A}^{-}}$ and backward ${\overline{\mathbf{E}} }_{b{A}^{-}}$ fields at the plane ${A}^{-}$ are obtained as

55 $${\overline{\mathbf{E}} }_{f{A}^{-}}=\frac{1}{2}\left(\overline{{\varvec{I}} }+\overline{{\varvec{I}} }\right){\overline{\mathbf{E}} }_{{A}^{-}}={\overline{\mathbf{E}} }_{{A}^{-}}.\quad {\overline{\mathbf{E}} }_{b{A}^{-}}=\frac{1}{2}\left(\overline{{\varvec{I}} }-\overline{{\varvec{I}} }\right){\overline{\mathbf{E}} }_{{A}^{-}}=0$$

Therefore, we have

56 $${\overline{\mathbf{E}} }_{f{A}^{-}}^{ }={\overline{\mathbf{E}} }_{{A}^{-}}^{ }={\overline{\mathbf{E}} }_{{A}^{+}}^{ }=2{\left(\overline{{\varvec{I}} }+{\overline{{\varvec{Z}}} }_{{A}^{+}}^{-1}\right)}^{-1}{\overline{\mathbf{E}} }_{f{A}^{+}}$$

Considering that the applied field at the port $B$ is equal to ${\overline{\mathbf{E}} }_{fB}$, then the forward propagating field (towards $z$ direction) at the plane ${A}^{+}$ will become ${\overline{\mathbf{E}} }_{f{A}^{+}}={e}^{-{\varvec{\Gamma}}{\overline{d} }_{AB}}{\overline{\mathbf{E}} }_{fB}$, hence

57 $${\overline{\mathbf{E}} }_{f{A}^{-}}^{ }=2{\left(\overline{{\varvec{I}} }+{\overline{{\varvec{Z}}} }_{{A}^{+}}^{-1}\right)}^{-1}{e}^{-{\varvec{\Gamma}}{\overline{d} }_{AB}}{\overline{\mathbf{E}} }_{fB}$$

Then, the transmission coefficient matrix is calculated as

58 $${{\varvec{S}}}_{21}=\frac{{\overline{\mathbf{E}} }_{f{A}^{-}}^{ }}{{\overline{\mathbf{E}} }_{fB}^{ }}=2{\left(\overline{{\varvec{I}} }+{\overline{{\varvec{Z}}} }_{{A}^{+}}^{-1}\right)}^{-1}{e}^{-{\varvec{\Gamma}}{\overline{d} }_{AB}}$$

The first element of the matrix ${{\varvec{S}}}_{21}$ denotes the transmission coefficient of the fundamental mode, if the modes are ordered properly.

**Figure 4:**
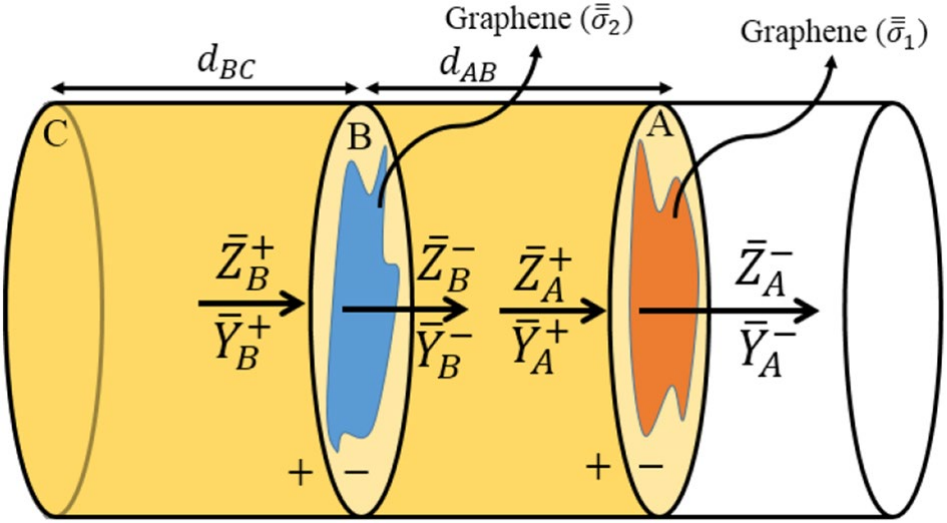
Three-layered structure with graphene plates at the interfaces.

As shown in Fig. 4, there are two layers of graphene inside the rectangular waveguide. The corresponding transmission coefficient is derived similarly. Assume that the applied field to the port C is ${\overline{\mathbf{E}} }_{fC}$, then the forward electric field at the plane ${B}^{+}$ is calculated as ${\overline{\mathbf{E}} }_{f{B}^{+}}^{ }={e}^{-{\varvec{\Gamma}}{\overline{d} }_{BC}}{\overline{\mathbf{E}} }_{fC}$. Considering the continuity of the tangential electric field at the plane B, we have

59 $${\overline{\mathbf{E}} }_{{B}^{+}}^{ }={\overline{\mathbf{E}} }_{{B}^{-}}^{ }={\overline{\mathbf{E}} }_{{B}^{ }}^{ }=2{\left(\overline{{\varvec{I}} }+{\overline{{\varvec{Z}}} }_{{B}^{+}}^{-1}\right)}^{-1}{\overline{\mathbf{E}} }_{f{B}^{+}}$$

Therefore, the forward and backward fields at the plane ${B}^{-}$ are obtained as

60 $${\overline{\mathbf{E}} }_{f{B}^{-}}=\frac{1}{2}\left(\overline{{\varvec{I}} }+{\overline{{\varvec{Z}}} }_{{B}^{-}}^{-1}\right){\overline{\mathbf{E}} }_{{B}^{-}}.\quad {\overline{\mathbf{E}} }_{b{B}^{-}}=\frac{1}{2}\left(\overline{{\varvec{I}} }-{\overline{{\varvec{Z}}} }_{{B}^{-}}^{-1}\right){\overline{\mathbf{E}} }_{{B}^{-}}$$

The forward field at the plane ${A}^{+}$ is written as

61 $${\overline{\mathbf{E}} }_{f{A}^{+}}^{ }={e}^{-{\varvec{\Gamma}}{\overline{d} }_{AB}}{\overline{\mathbf{E}} }_{f{B}^{-}}$$

Consequently, using Eq. (53), the total electric field at the plane ${A}^{+}$ can be written as

62 $${\overline{\mathbf{E}} }_{{A}^{+}}=2{\left(\overline{{\varvec{I}} }+{\overline{{\varvec{Z}}} }_{{A}^{+}}^{-1}\right)}^{-1}{\overline{\mathbf{E}} }_{f{A}^{+}}$$

Considering the continuity of the electric field components at the plane ${A}$ we have

63 $${\overline{\mathbf{E}} }_{{A}^{-}}={\overline{\mathbf{E}} }_{{A}^{+}}={\overline{\mathbf{E}} }_{A}$$

Since the electric field at the plane ${A}^{-}$ is completely propagating forward, from Eqs. (61)–(63) we can write

64 $${\overline{\mathbf{E}} }_{f{A}^{-}}={\overline{\mathbf{E}} }_{{A}^{-}}={\overline{\mathbf{E}} }_{{{A}^{+}}}=2{\left(\overline{{\varvec{I}} }+{\overline{{\varvec{Z}}} }_{{A}^{+}}^{-1}\right)}^{-1}{e}^{-{\varvec{\Gamma}}{\overline{d} }_{AB}}{\overline{\mathbf{E}} }_{f{B}^{-}}$$

By substitution of Eqs. (60) and (52) in (64)

65 $$ \begin{aligned} {\overline{\mathbf{E}}}_{{fA^{ - } }}^{ } & = 2\left( {\overline{\user2{I}} + \overline{\user2{Z}}_{{A^{ + } }}^{ - 1} } \right)^{ - 1} e^{{ - {{\varvec{\Gamma}}}\overline{d}_{AB} }} {\overline{\mathbf{E}}}_{{fB^{ - } }}^{ } = 2\left( {\overline{\user2{I}} + \overline{\user2{Z}}_{{A^{ + } }}^{ - 1} } \right)^{ - 1} e^{{ - {{\varvec{\Gamma}}}\overline{d}_{AB} }} \times \frac{1}{2}\left( {\overline{\user2{I}} + \overline{\user2{Z}}_{{B^{ - } }}^{ - 1} } \right){\overline{\mathbf{E}}}_{{B^{ - } }}^{ } \\ & = 2\left( {\overline{\user2{I}} + \overline{\user2{Z}}_{{A^{ + } }}^{ - 1} } \right)^{ - 1} e^{{ - {{\varvec{\Gamma}}}\overline{d}_{AB} }} \times \frac{1}{2}\left( {\overline{\user2{I}} + \overline{\user2{Z}}_{{B^{ - } }}^{ - 1} } \right) \times 2\left( {\overline{\user2{I}} + \overline{\user2{Z}}_{{B^{ + } }}^{ - 1} } \right)^{ - 1} {\overline{\mathbf{E}}}_{{fB^{ + } }}^{ } \\ & = 2\left( {\overline{\user2{I}} + \overline{\user2{Z}}_{{A^{ + } }}^{ - 1} } \right)^{ - 1} e^{{ - {{\varvec{\Gamma}}}\overline{d}_{AB} }} \times \frac{1}{2}\left( {\overline{\user2{I}} + \overline{\user2{Z}}_{{B^{ - } }}^{ - 1} } \right) \times 2\left( {\overline{\user2{I}} + \overline{\user2{Z}}_{{B^{ + } }}^{ - 1} } \right)^{ - 1} e^{{ - {{\varvec{\Gamma}}}\overline{d}_{BC} }} {\overline{\mathbf{E}}}_{fC}^{ } \\ \end{aligned} $$

As a result, from Eq. (65), the transmission coefficient is obtained as

66 $${{\varvec{S}}}_{21}=\frac{{\overline{\mathbf{E}} }_{f{A}^{-}}}{{\overline{\mathbf{E}} }_{fC}}=2{\left(\overline{{\varvec{I}} }+{\overline{{\varvec{Z}}} }_{{A}^{+}}^{-1}\right)}^{-1}{e}^{-{\varvec{\Gamma}}{\overline{d} }_{AB}}\times \frac{1}{2}\left(\overline{{\varvec{I}} }+{\overline{{\varvec{Z}}} }_{{B}^{-}}^{-1}\right)\times 2{\left(\overline{{\varvec{I}} }+{\overline{{\varvec{Z}}} }_{{B}^{+}}^{-1}\right)}^{-1}{e}^{-{\varvec{\Gamma}}{\overline{d} }_{BC}}$$

**Figure 5:**
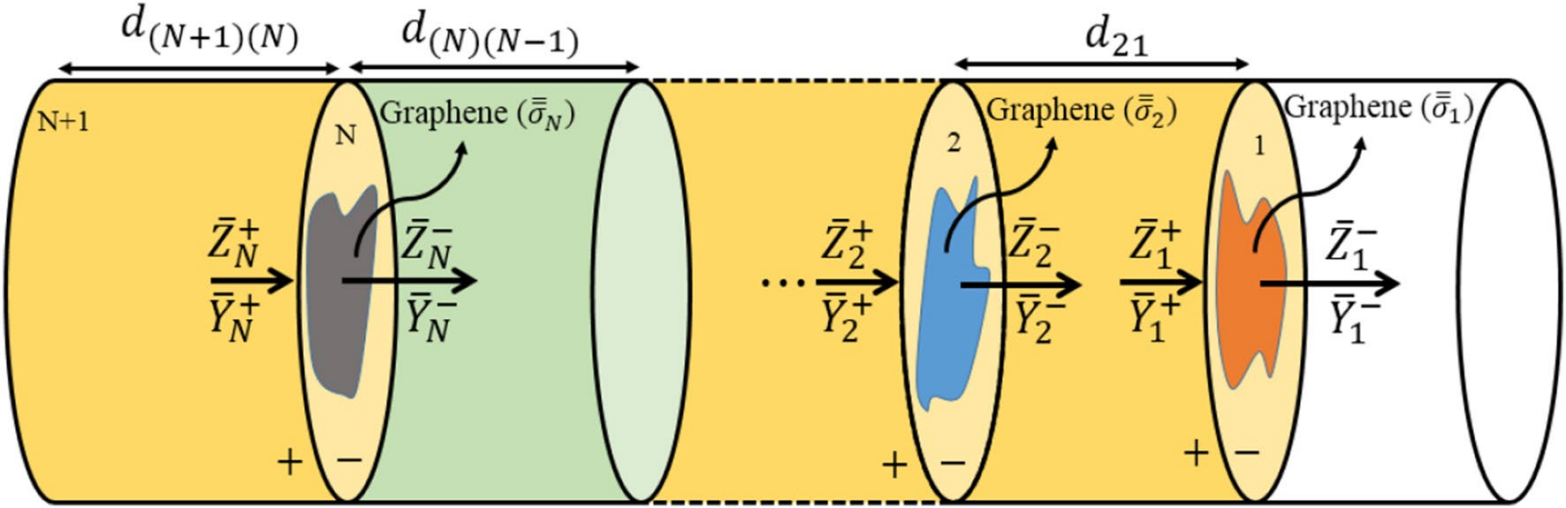
($N+1$)-layered 3D graphene-loaded multilayered structure.

Hence, in general, for an $(N+1)$-layered structure shown in Fig. 5, the transmission coefficients can be written as follows

67 $$ \begin{aligned} {\varvec{S}}_{21} & = \frac{{{\overline{\mathbf{E}}}_{{f1^{ - } }}^{ } }}{{{\overline{\mathbf{E}}}_{{f\left( {N + 1} \right)}}^{ } }} = 2\left( {\overline{\user2{I}} + \overline{\user2{Z}}_{{1^{ + } }}^{ - 1} } \right)^{ - 1} e^{{ - {{\varvec{\Gamma}}}\overline{d}_{21} }} \times \frac{1}{2}\left( {\overline{\user2{I}} + \overline{\user2{Z}}_{{2^{ - } }}^{ - 1} } \right) \times 2\left( {\overline{\user2{I}} + \overline{\user2{Z}}_{{2^{ + } }}^{ - 1} } \right)^{ - 1} e^{{ - {{\varvec{\Gamma}}}\overline{d}_{32} }} \\ & \quad \quad \quad \quad \quad \quad \quad \quad \quad \quad \quad \quad \vdots \\ & \quad \times \frac{1}{2}\left( {\overline{\user2{I}} + \overline{\user2{Z}}_{{\left( {N - 1} \right)^{ - } }}^{ - 1} } \right) \times 2\left( {\overline{\user2{I}} + \overline{\user2{Z}}_{{\left( {N - 1} \right)^{ + } }}^{ - 1} } \right)^{ - 1} e^{{ - {{\varvec{\Gamma}}}\overline{d}_{{\left( N \right)\left( {N - 1} \right)}} }} \\ & \quad \times \frac{1}{2}\left( {\overline{\user2{I}} + \overline{\user2{Z}}_{{N^{ - } }}^{ - 1} } \right) \times 2\left( {\overline{\user2{I}} + \overline{\user2{Z}}_{{N^{ + } }}^{ - 1} } \right)^{ - 1} e^{{ - {{\varvec{\Gamma}}}\overline{d}_{{\left( {N + 1} \right)\left( N \right)}} }} \\ \end{aligned} $$

Note that the scattering matrices ${{\varvec{S}}}_{11}$ and ${{\varvec{S}}}_{21}$ are square matrices of size $2{N}_{\varphi }{N}_{r}\times 2{N}_{\varphi }{N}_{r}$. The entry in the $i$ th row and the $j$ th column, i.e.${S}_{11}^{ij}/{S}_{21}^{ij}$, represents the reflection/transmission coefficient of $i$ th mode when $j$ th mode is incident on the input port. For example, the entry $\left(\mathrm{1,1}\right)$ denotes that the excitation mode in the input port is the first mode, i.e. the fundamental ${\mathrm{TE}}_{11}$ mode in a circular waveguide, and the reflected/ transmitted mode is the same mode $({\mathrm{TE}}_{11})$. The entry $\left(\mathrm{2,1}\right)$ denotes that the excitation mode in the input port is the first mode $({\mathrm{TE}}_{11})$ and the reflected/transmitted mode is the second mode of the circular waveguide, i.e. ${\mathrm{TM}}_{01}$ mode. In this paper the reflection and transmission coefficients for the entry $\left(\mathrm{1,1}\right)$, i.e. both incident and reflected modes being the fundamental mode, are plotted which pertains to the ${\mathrm{TE}}_{11}$ mode in the circular waveguide and the TEM mode in the coaxial waveguide.

## Validation of the proposed method

To validate the proposed method, two multilayer graphene-loaded structures are examined in the cylindrical coordinates system: a circular waveguide and a coaxial cable. Both structures are analyzed for different values of graphene chemical potential and varying numbers of graphene plates. The results of the proposed method and CST simulations are compared to each other.

### Analysis of a graphene-loaded circular waveguide

**Figure 6:**
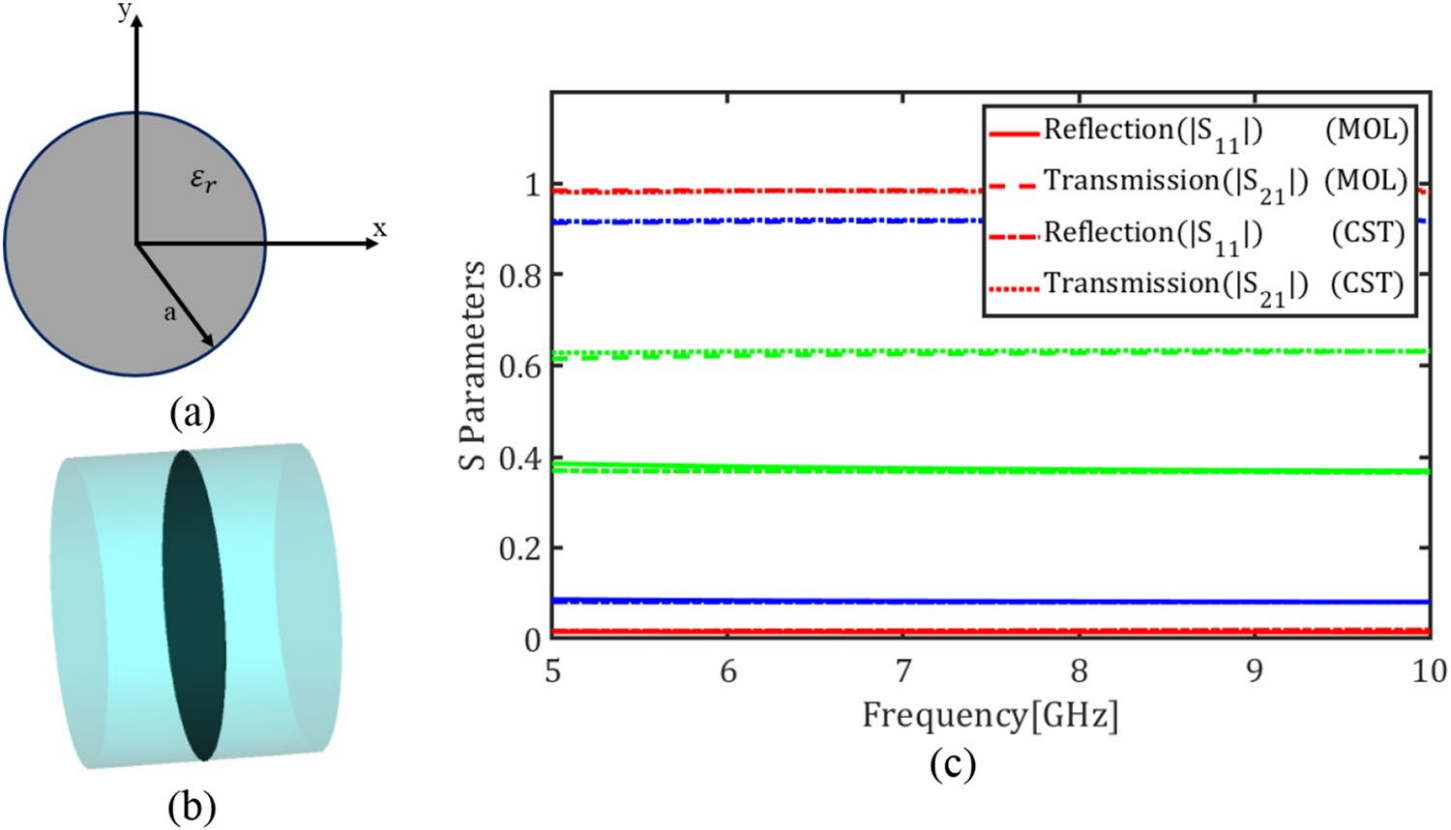
(**a**) Cross section of the circular waveguide, (**b**) The waveguide loaded with a graphene plate, (**c**) Magnitude of transmission and reflection coefficients of the fundamental mode for different values of the chemical potential (red) ${\mu }_{c}$= 0.05 eV, (blue) ${\mu }_{c}$= 0.3 eV and (Green) ${\mu }_{c}$= 2 eV using E-MOL analysis and CST simulations.

In this section, as an example of a graphene-loaded multilayer structure, graphene plates are placed inside a circular waveguide. The cross-section of the waveguide is shown in Fig. 6a. The radius of the waveguide is $a = 10$ mm which is filled with a dielectric permittivity of ${\varepsilon }_{r}=60$. Figure 6b depicts the circular waveguide loaded with a graphene plate. For THz and sub-THz regimes in the Kubo formalism of graphene the term pertaining to the intraband electron tramsitions dominates and thus the surface conductivity reduces to a Drude model^14^. In this model the relaxation time $\tau = 0.1 \mathrm{ps}$, and magnetic bias intensity ${B}_{0}=0$ are assumed as graphene parameters. Note that the conductivity of an unmagnetized graphene plate will become a diagonal tensor with equal diagonal entries^7^.

Figure 6c depicts the reflection coefficient $\left|{S}_{11}\right|$ and transmission coefficient $\left|{S}_{21}\right|$ of the fundamental mode of the circular waveguide, i.e. TE_11_, for the graphene chemical potentials ${\mu }_{c}=0.05$, ${\mu }_{c}=0.3$, ${\mu }_{c}=1.2$ and ${\mu }_{c}=2$ eV. The values $\left|{S}_{11}\right|$ and $\left|{S}_{21}\right|$ denote the electric field ratios at the ports of the waveguide. It is evident that ${\left|{S}_{11}\right|}^{2}$, ${\left|{S}_{21}\right|}^{2}$, and $1-{\left|{S}_{11}\right|}^{2}-{\left|{S}_{21}\right|}^{2}$ represent the reflectance, transmittance, and absorptance, respectively, which are power ratios and can be easily calculated from Fig. 6c. The reflectance is due to the discontinuity imposed by the graphene boundary condition and the absorptance is due to the lossy nature of the graphene.

**Figure 7:**
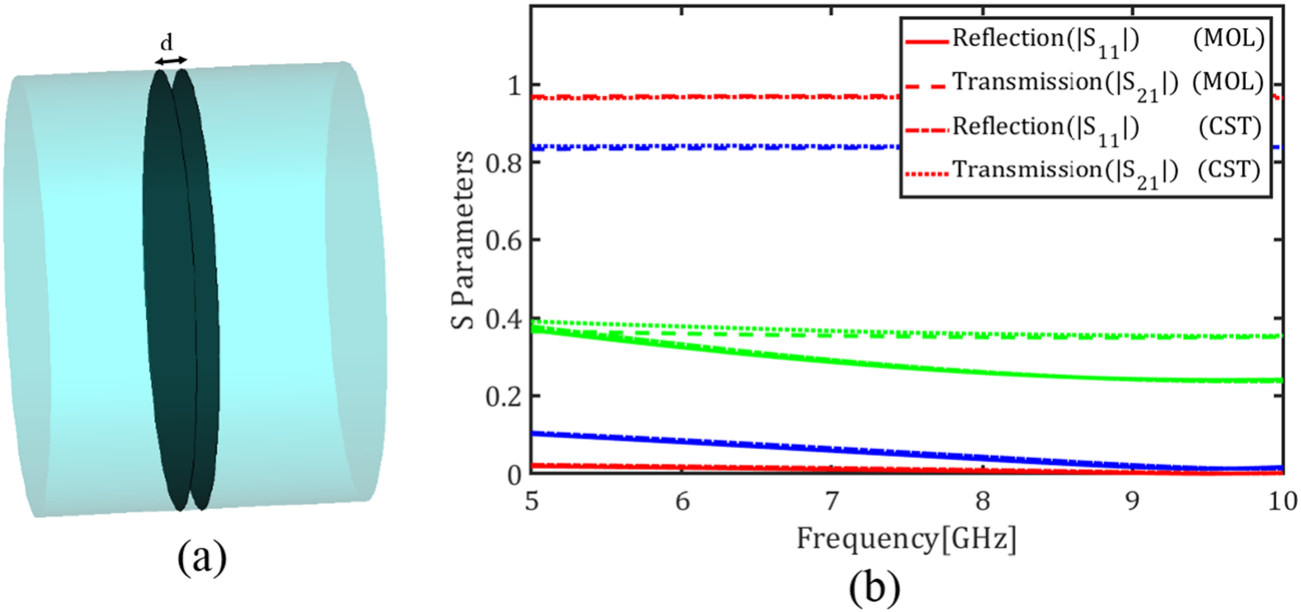
(**a**) A circular waveguide loaded with two graphene plates, (**b**) Magnitude of transmission and reflection coefficients of the fundamental mode for different values of the chemical potential (red) ${\mu }_{c}$= 0.05 eV, (blue) ${\mu }_{c}$= 0.3 eV and (green) ${\mu }_{c}$= 2 eV using E-MOL analysis and CST simulations.

**Figure 8:**
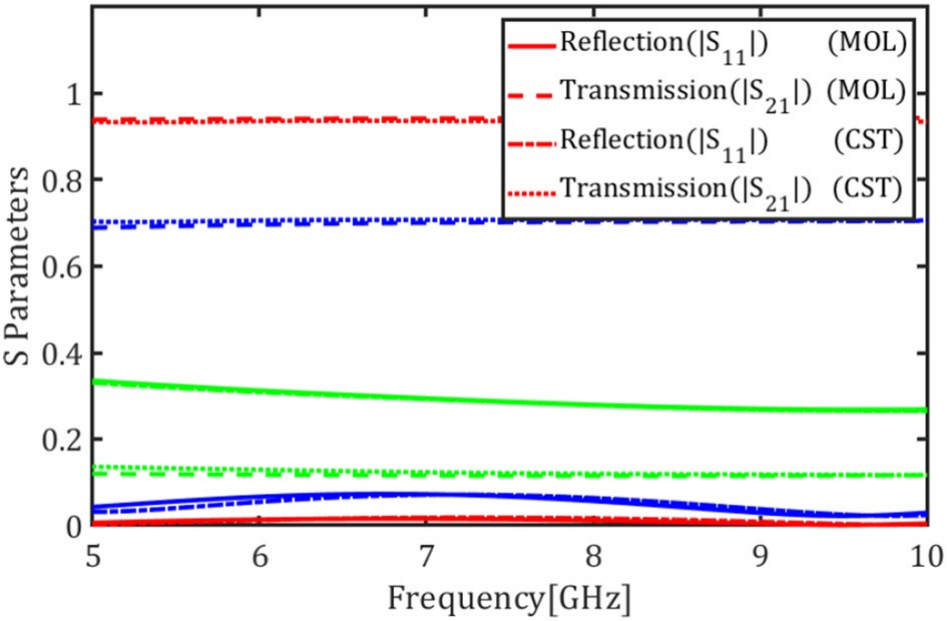
Magnitude of transmission and reflection coefficients of the fundamental mode in a circular waveguide loaded with four graphene plates for different values of the chemical potential (red) ${\mu }_{c}$= 0.05, (blue) ${\mu }_{c}$ = 0.3 and (green) ${\mu }_{c}$= 2 eV using E-MOL analysis and CST simulations.

Figure 7a depicts two graphene plates with a spacing $d = 1 \mathrm{mm}$ inside a circular waveguide while Fig. 7b shows the transmission and reflection coefficients of the fundamental mode for the chemical potentials ${\mu }_{c}=0.05$, ${\mu }_{c}=0.3$ and ${\mu }_{c}=2$ eV. Comparing Figs. 6c and 7b denotes that adding the second graphene plate decreases the transmission coefficient and introduces frequency dispersion in the reflection coefficient. Finally Fig. 8 shows the transmission and reflection coefficients of the circular waveguide loaded with four graphene plates with the spacings $d = 1 \mathrm{mm}$ for the chemical potentials ${\mu }_{c}=0.05$, ${\mu }_{c}=0.3$ and ${\mu }_{c}=2$ eV.

Table 1 presents the time required for the computation of the reflection and transmission coefficients using the E-MoL (implemented by MATLAB) and the CST full wave simulation software. Both methods are implemented under the same conditions exploiting an Intel Core i7 8700K CPU, 32 GB RAM desktop computer. In the data provided by Table 1 the chemical potential is assumed to be ${\mu }_{c}=0.3$ eV. As expected, the semi-analytical E-MoL technique is much more efficient than the full wave CST simulation software.

**Table 1:** The simulation times (in seconds) for E-MOL and CST for the chemical potential ${\mu }_{c}=$ 0.3 eV.

Number of graphene plates	E-MOL	CST
One (Fig. 6c)	0.29	562
Two (Fig. 7b)	0.33	589
Four (Fig. 8)	0.34	616

### Analysis of a graphene-loaded coaxial cable

**Figure 9:**
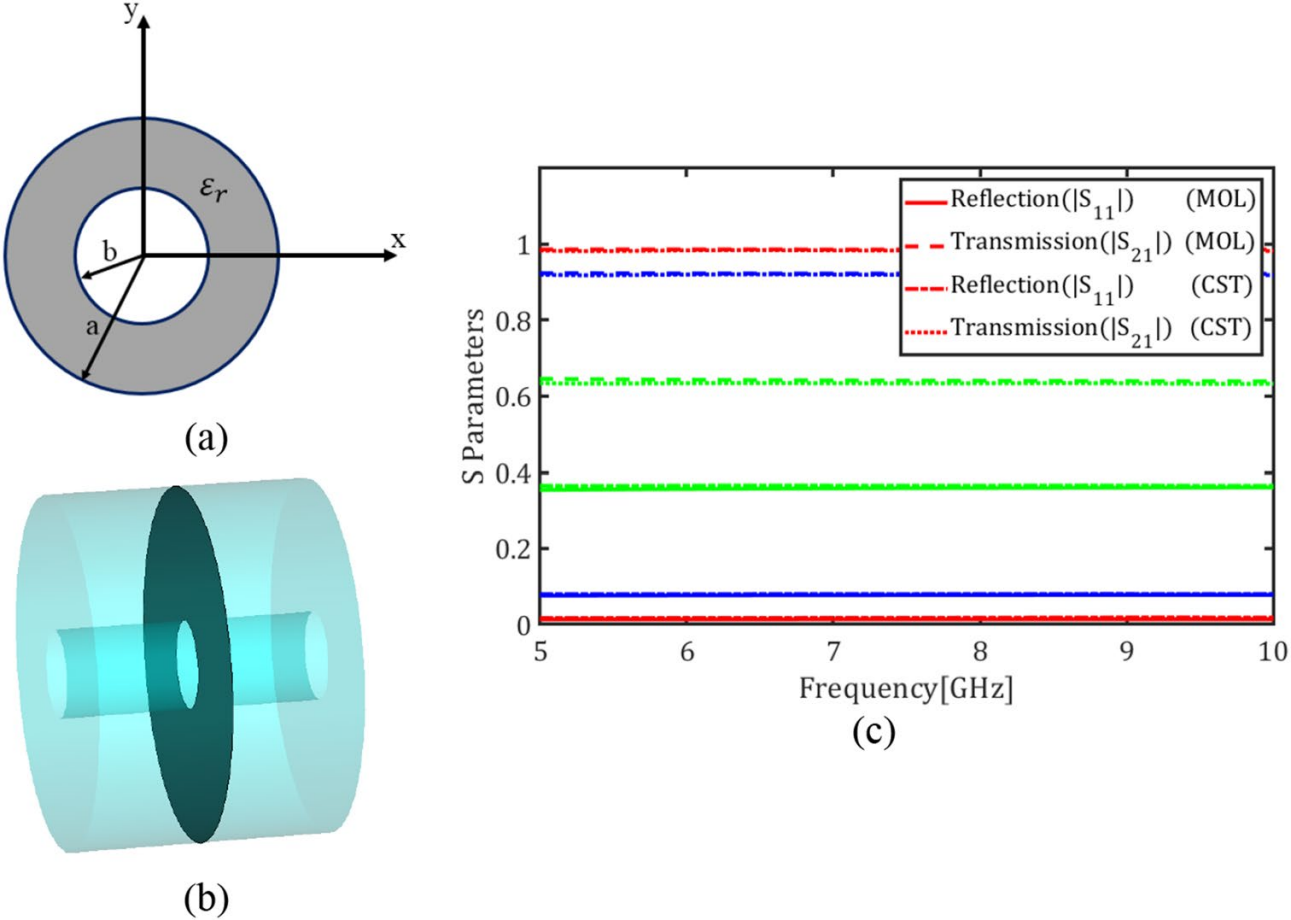
(**a**) Cross section of the coaxial cable structure, (**b**) a graphene plate inside the coaxial cable, (**c**) magnitude of transmission and reflection coefficients of the fundamental mode for different values of the chemical potential (red) ${\mu }_{c}$ = 0.05, (blue) ${\mu }_{c}$ = 0.3 and (Green) ${\mu }_{c}$= 2 eV using E-MOL analysis and CST simulations.

In this section the graphene plates are placed inside the coaxial cable and the structure is analyzed in terms of the chemical potential and the number of graphene plates. The cross-section of the coaxial cable is shown in Fig. 9a. The outer and inner radi are $a=10$ and $b=2.5$ mm, respectively, and the cable is filled with the permittivity ${\varepsilon }_{r}$= 60. Figure 9b shows the coaxial cable loaded with a single annular graphene plate. Figure 9c shows the transmission and reflection coefficients of the fundamental mode, i.e. TEM mode, for different values of the graphene chemical potential ${\mu }_{c}=0.05$, ${\mu }_{c}=0.3$ and ${\mu }_{c}=2$ eV.

**Figure 10:**
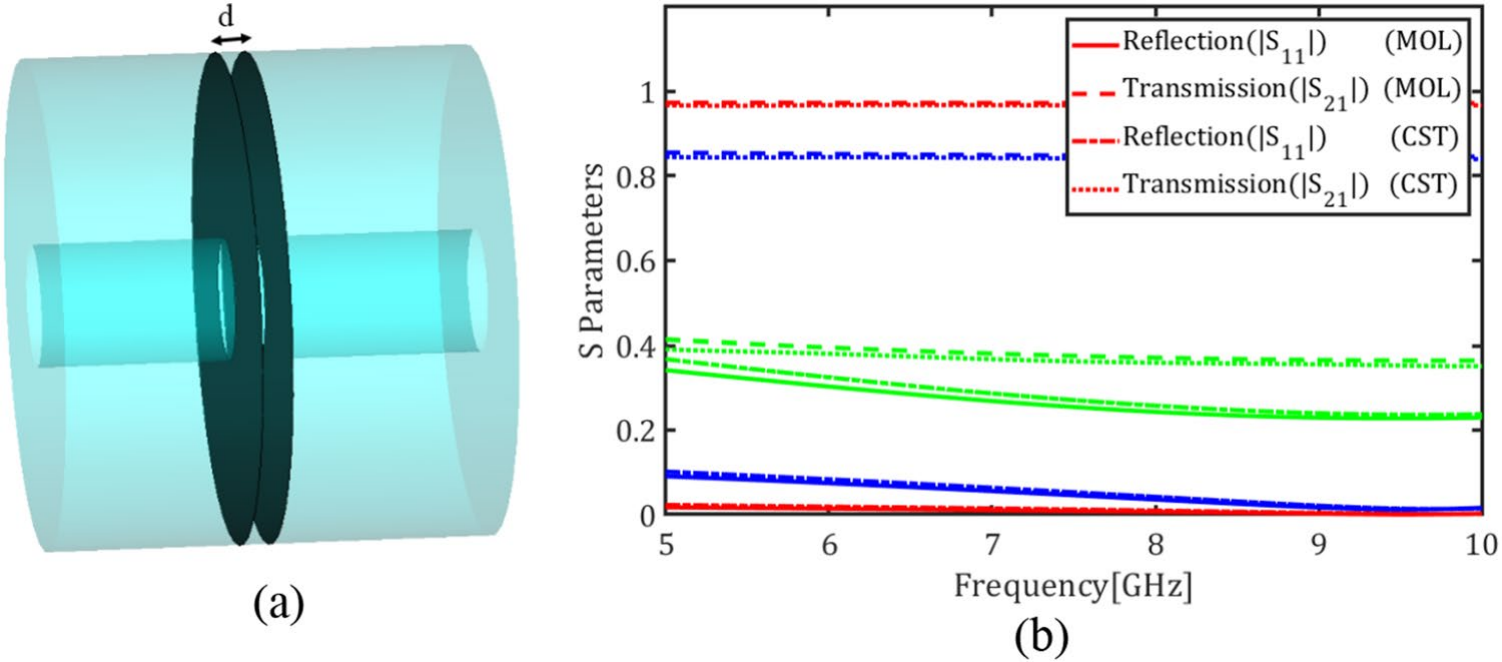
(**a**) The coaxial cable loaded with two graphene plates, (**b**) magnitude of transmission and reflection coefficients of the fundamental mode for different values of the chemical potential (red) ${\mu }_{c}$= 0.05, (blue) ${\mu }_{c}$= 0.3, and (green) ${\mu }_{c}$= 2 eV using E-MOL analysis and CST simulations.

**Figure 11:**
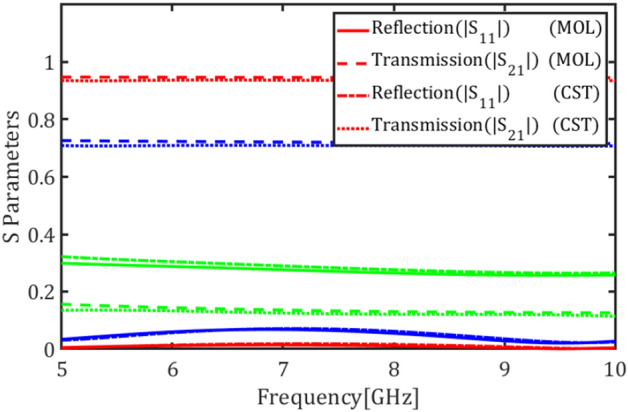
Magnitude of transmission and reflection coefficients of the coaxial cable loaded with four graphene plates for the fundamental mode for different values of the chemical potential (red) ${\mu }_{c}$= 0.05, (blue) ${\mu }_{c}$= 0.3 and (green) ${\mu }_{c}$= 2 eV using E-MOL analysis and CST simulations.

Figure 10a shows the coaxial cable loaded with two graphene plates with a spacing of $d$ = 1 mm. Figure 10b depicts the transmission and reflection coefficients of the cable for different values of the graphene chemical potential ${\mu }_{c}=0.05$, ${\mu }_{c}=0.3$, and ${\mu }_{c}=2$ eV. Finally, Fig. 11 shows the transmission and reflection coefficients of the cable loaded with four graphene plates with spacings $d$ = 1 mm for different values of the graphene chemical potential ${\mu }_{c}=0.05$, ${\mu }_{c}=0.3$, and ${\mu }_{c}=2$ eV.

Considering the reflection and transmission coefficient plots, increasing the chemical potential increases the graphene conductivity and thus reduces its transparency which results in an increase/decrease in the reflection/transmission coefficient, respectively. In other words, the reflected and transmitted powers of the waveguide can be tuned by an electric voltage controlling the graphene plate. This finds applications in tunable microwave waveguide attenuators. Also, this can be utilized in phased array antennas to control the feeding amplitude of each element of the antenna array. Note that no resonant behavior is observed in the reflection and transmission coefficients because the resonance frequency of a graphene plate is far beyond the microwave frequencies. This nearly flat response offers wideband microwave devices.

## Conclusion

In this paper, an extended method of lines (E-MoL) is presented for the analysis of graphene-loaded multilayer structures in the cylindrical coordinates. To do so, first, the impedance and admittance transformations imposed by graphene plates are obtained in the cylindrical coordinates system. Having the impedance and admittance transformations, the impedance and admittance matrices can be calculated in each plane of the structure. Then from the input impedance of the structure the reflection coefficient is derived. Finally by transferring the fields from the input to the output of the multilayered stack, the transmission coefficient is computed. To validate the proposed method, a circular waveguide and a coaxial cable loaded with graphene plates are analyzed and the results are compared with the CST full wave simulations. The proposed theory has the potential to model multilayered absorbers and radiating structures accurately and efficiently which is a topic of the future research. The MoL can be generalized to model complicated boundary conditions other than graphene in planar and quasi-planar waveguide structures in integrated microwave and optical circuits and also in circular and conformal antennas. The graphene-loaded optical fibers are attractive optical devices in which the dispersive properties of graphene emerges and can be characterized using the E-MoL preseted in this paper.

## Data Availability

The datasets generated and analyzed during the current study are available from the corresponding author on reasonable request.
