# Author Correction: The method of lines extension for the analysis of multilayered graphene-loaded structures in cylindrical coordinates

**DOI:** 10.1038/s41598-023-35756-7

**Published:** 2023-05-31

**Authors:** Ali Mehrdadian, Keyvan Forooraghi, Mehri Ziaee Bideskan

**Affiliations:** Department of Electrical and Computer Engineering, Tarbiat Modares University, Tehran, 14115-194 Iran

Correction to: *Scientific Reports* 10.1038/s41598-022-17016-2, published online 26 July 2022

The original version of this Article contained errors.

Ali Mehrdadian was incorrectly affiliated with ‘Iran National Science Foundation (INSF), Tehran, Iran’.

The correct affiliation is listed below.

Department of Electrical and Computer Engineering, Tarbiat Modares University, Tehran, 14115-194, Iran.

In addition, the Funding section was omitted.

It now reads:

“The work is financially supported by ‘Iran National Science Foundation, (INSF), Tehran, Iran’, under contract number 99001238.”

The original Article has been corrected.

